# An Augmented Reality Audio-Motor Training Game for Improving Speech-in-Noise Perception: Single-Arm Pilot Feasibility Study

**DOI:** 10.2196/91260

**Published:** 2026-05-14

**Authors:** Pooseung Koh, Inyong Choi, Hyo-jeong Lee, Sungyoung Kim

**Affiliations:** 1 Graduate School of Culture Technology, Korea Advanced Institute of Science and Technology, Yuseong-gu Daehakro 291, Daejeon, Daejeon, 34141, Republic of Korea, 82 01027537697; 2 Department of Communication Sciences and Disorderes, University of Iowa, Iowa City, IA, United States; 3 Laboratory of Hearing, Balance and Integrated Neuroscience, College of Medicine, Hallym University, Anyang, Gyeongi-do, Republic of Korea; 4 Department of Otorhinolaryngology-Head and Neck Surgery, College of Medicine, Hallym University, Chuncheon, Gangwon-do, Republic of Korea

**Keywords:** augmented reality, auditory training, selective auditory attention, gamification, mobile health, mHealth

## Abstract

**Background:**

Difficulty understanding speech in noisy environments is a primary challenge of hearing impairment, inadequately addressed by hearing aids alone. While auditory training can enhance selective attention and speech perception, current digital programs face poor user adherence and lack realistic 3D spatial audio.

**Objective:**

This pilot study evaluated the feasibility, usability, and preliminary efficacy of ARIA (Augmented Reality Immersive Auditory training), a handheld mobile intervention that provides gamified at-home auditory training to middle-aged adults via earbud-delivered spatial audio.

**Methods:**

In this single-arm, pre-post–follow-up pilot study, 11 adults (mean age 53.0, SD 3.0 y) with functional hearing not requiring amplification completed a 4-week at-home training program using ARIA on provided devices (iPhone 14 Pro, AirPods Pro 2). Speech-in-noise perception was assessed via the Korean Matrix Sentence Test at baseline, 4 weeks, and 8 weeks at 3 signal-to-noise ratios (SNRs; 0 dB, −6 dB, and −9 dB, respectively). Feasibility, usability (System Usability Scale), user experience (Player Experience of Need Satisfaction), in-game performance, and qualitative feedback were collected.

**Results:**

Protocol completion was 100% (11/11), demonstrating technical feasibility. Exploratory efficacy analyses revealed statistically significant speech-in-noise improvements posttraining across all conditions (0 dB: *t*_10_=3.43, *P*=.02; −6 dB: *t*_10_=5.34, *P*<.001; −9 dB: *t*_10_=4.34*, P*=.004). Gains were maintained at the 8-week follow-up. In-game localization improvements correlated significantly with speech perception gains at −6 dB SNR (ρ=0.639; *P*=.03) and −9 dB SNR (ρ=0.612; *P*=.045). User experience showed mixed results: the mean System Usability Scale score was 70.2 (SD 19.6; range 47.5‐92.5), reflecting substantial individual differences in usability perception. While 72% (n=8) reported difficulties with the augmented reality (AR) environmental setup, 63% reported genuine mastery-driven engagement with core gameplay. Thematic analysis revealed a dissociation between peripheral usability challenges (setup friction, “homework” characterization due to protocol structure) and successful engagement with the training paradigm itself.

**Conclusions:**

This pilot demonstrated the feasibility of AR-based audio-motor training for at-home delivery and revealed encouraging preliminary efficacy signals, warranting progression to controlled efficacy trials. Formative findings identified specific usability refinements needed for broader implementation, particularly streamlining AR setup while preserving the core gameplay elements that successfully fostered competence and engagement. These insights provide clear guidance for platform optimization and randomized controlled trial design.

## Introduction

### Growing Challenge of Hearing Loss and the Need for Auditory Training

Age-related hearing loss affects 466 million people worldwide, a number projected to reach 630 million by 2030, and represents the fourth leading cause of disability, as well as one of the largest modifiable risk factors for dementia [1-5]. A primary challenge for individuals with hearing impairment is understanding speech in noisy environments, which demands a complex interplay between sensory input and cognitive processing [6,7]. While hearing aids restore audibility, they inadequately address the cognitive demands of listening in noise [8], highlighting a critical need for auditory training interventions that enhance selective auditory attention—the ability to focus on target sounds amidst distractors, exemplified by the “cocktail party effect” [9]. Neuroscientific research using functional magnetic resonance imaging and electroencephalogram confirms that specific neural networks dedicated to selective attention are critical for both sound localization and speech segregation, and that targeted training can improve these functions in hearing-impaired individuals [10-14]. Systematic reviews demonstrate that auditory training paradigms can enhance auditory and cognitive skills, including speech perception, working memory, attention, and processing speed [15-17]. However, traditional programs face significant barriers including poor user adherence and limited accessibility [18]. Digital platforms like LACE and Amptify have improved accessibility through at-home delivery [19,20]. However, an opportunity to incorporate spatial audio cues was identified—a dimension absent from current programs but fundamental to inherently 3D, real-world listening. Real-world listening is inherently 3D, with the brain leveraging binaural cues to localize and segregate sound sources, primarily interaural time differences (microsecond delays between ears) and interaural level differences (intensity differences between ears) [21].

### Audio-Motor Training

Beyond acoustic properties, the listener’s state of engagement critically modulates auditory processing and learning [22]. Research demonstrates that “active listening”—the cognitive engagement with sound—can enhance auditory performance, with studies showing that active listening postures improve 3D sound localization and that embodied training activities can benefit related perceptual skills [23,24]. This engagement often manifests through spontaneous head movements, which dynamically alter interaural time difference and interaural level difference cues to improve localization and resolve spatial ambiguities [25]. Studies in virtual reality (VR) environments suggest that listeners increase head movements when facing challenging listening conditions, reflecting this as an instinctual learning strategy [25,26]. Building on this, research by Valzolgher et al [27] supports the use of active, sensorimotor training to improve spatial auditory performance. While the evidence for immediate transfer to speech-in-noise remains an area of ongoing investigation, a closed-loop audio-motor game requiring continuous sensorimotor prediction and correction has been shown to yield a 25% improvement in speech-in-noise perception [28]. This suggests that such paradigms may achieve generalized learning that transfers beyond trained stimuli, potentially addressing the specificity limits common in traditional training [28]. Therefore, our system design integrates active listening tasks, natural head movements via 6-degree-of-freedom tracking, and goal-directed audio-motor interaction to explore whether these mechanisms can support robust and generalizable auditory learning.

### Augmented Reality and Gamification

Translating embodied audio-motor training from the laboratory to daily life requires technology that is both environmentally aware and accessible. Modern augmented reality (AR) frameworks are a promising modality for this challenge, having become increasingly accessible through advances in mobile hardware and software [29]. While previous work has explored audio-motor feedback on tablets or in VR [27-30], AR provides the critical advantage of integrating virtual elements into the user’s real environment rather than replacing it. We developed ARIA (Augmented Reality Immersive Auditory training), a handheld mobile intervention that enables in situ training, where the system acoustically adapts to the participant’s actual room geometry. By tracking smartphone position and using earbud-embedded gyroscopes for head rotation, ARIA delivers spatial audio that facilitates natural, embodied training within users’ physical spaces. While serious games show mixed results, well-designed gamification incorporating clear goals, immediate feedback, and structured progression can address adherence challenges [31,32]. Following these principles, our system was refined from an earlier prototype that informed the initial design [33], synthesizing evidence-based audio-motor training with AR’s environmental mapping to create an engaging, acoustically realistic training framework.

### Study Objectives

The primary aims of this pilot study were to (1) evaluate the feasibility and acceptability of delivering AR-based auditory training to a foundational cohort of middle-aged adults (aged 50‐65 y) to establish platform viability, (2) assess usability through standardized scales and qualitative feedback, and (3) conduct exploratory analyses of speech-in-noise outcomes to inform future efficacy trials.

## Methods

### Study Design

This study was a single-arm, pre-post–follow-up pilot feasibility trial conducted over a period of 8 weeks, evaluating an at-home training protocol for ARIA. All participants provided written informed consent.

### Participants and Recruitment

The study recruited middle-aged adults aged 50 to 65 years, an at-risk demographic for age-related sensorineural hearing loss, via university and company email lists supplemented by snowball sampling. Inclusion criteria were as follows: (1) aged 50 to 65 years, (2) self-reported functional hearing not requiring amplification, (3) ability to commit to an 8-week protocol, and (4) Korean fluency. Exclusion criteria were as follows: (1) diagnosed hearing impairment requiring hearing aids, (2) history of ear surgery or chronic ear disease, (3) neurological conditions affecting auditory or cognitive processing, and (4) inability to complete touchscreen-based tasks. Following screening, 11 participants (8 males and 3 females) enrolled. This cohort was specifically selected to evaluate the technical feasibility of the ARIA interface in a relatively high-functioning group before extending the intervention to clinical populations with advanced hearing loss.

### Ethical Considerations

This study was reviewed and approved by the Institutional Review Board of the Korea Advanced Institute of Science and Technology (KAISTIRB-2025‐32). All participants provided written informed consent prior to enrollment. Participants were informed of the study’s purpose, procedures, potential risks, and their right to withdraw at any time without penalty. Personal data were anonymized using participant identification codes, and all data were stored on password-protected, encrypted servers accessible only to the research team. Participants received financial compensation (up to ₩300,000, approximately US $216) contingent on protocol adherence.

### The Intervention Delivery Method

The ARIA intervention was delivered as a handheld mobile app running on iOS devices. To ensure standardization across participants, all were provided with an iPhone 14 Pro and Apple AirPods Pro 2 earbuds. The app was developed using *Unity* 2022.3 LTS with ARKit 4.0 for spatial tracking and used the *Wwise* audio engine for 3D sound rendering [34,35].

#### Hardware Configuration

The iPhone 14 Pro’s Light Detection and Ranging sensor and ARKit capabilities enabled real-time environmental mapping and 6-degree-of-freedom position tracking. The AirPods Pro 2 provided spatial audio rendering with dynamic head tracking via embedded gyroscopes, updating audio presentation based on head orientation. This configuration allowed participants to experience naturalistic spatial cues while moving freely within their physical environment.

#### Gameplay and Training Protocol

**Figure 1.**
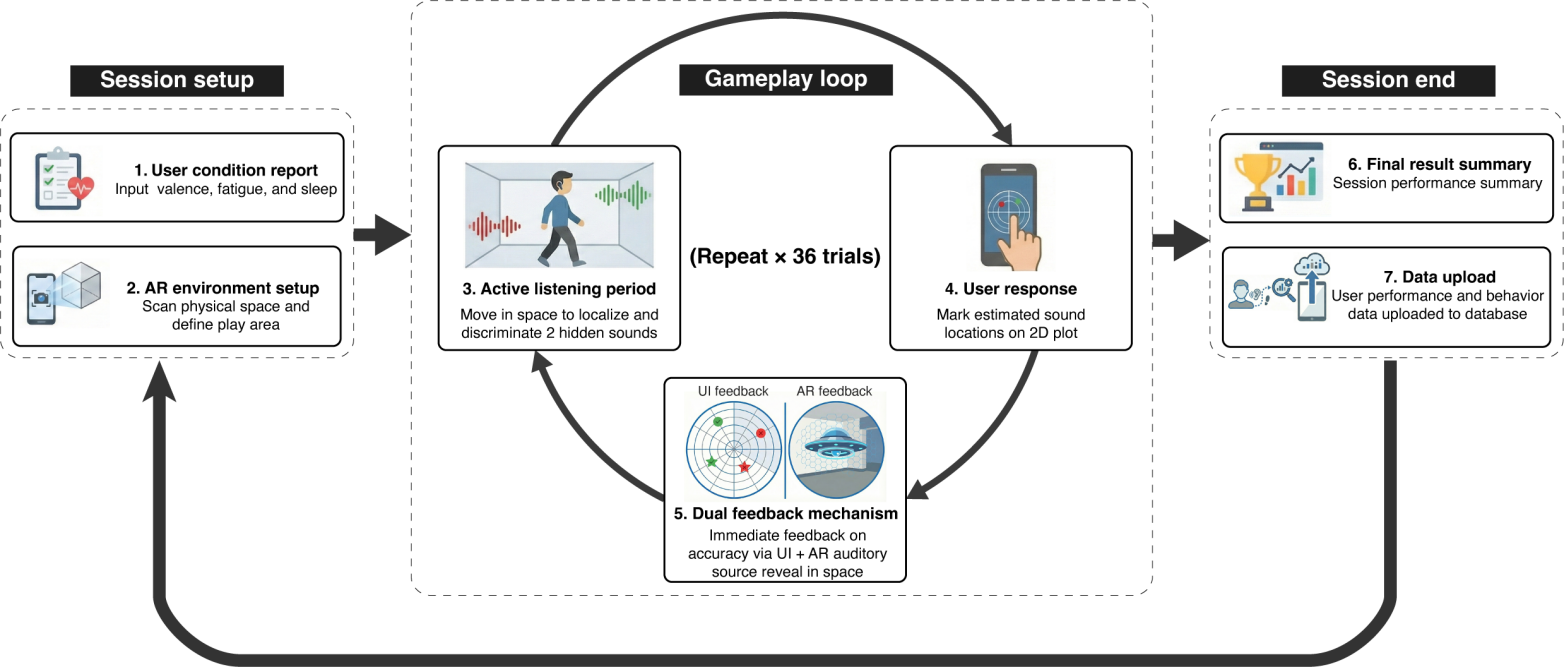
App flow diagram from the user’s perspective. Augmented Reality Immersive Auditory training (ARIA) session flow depicting setup, gameplay loop, and session end from the user’s perspective. AR: augmented reality; UI: user interface.

Each game session follows a structured workflow (Figure 1), beginning with participants reporting their current state, modeled after the Affective Digital Sliders (hours of sleep, fatigue 1‐100, and mood valence 1‐100) [36].

Before gameplay, participants scan their physical environment with the mobile device and designate a play area. The ARIA system analyzes the detected surfaces and applies acoustic reflectors to enable first-order early reflections through the *Wwise* audio engine. This environmental calibration ensures that the spatial audio matches the geometric properties of the user’s actual space, creating an in situ training experience. A video demonstration of all session phases, including environment setup, active listening, response interface, and feedback visualization, is provided in Multimedia Appendix 1.

**Figure 2.**
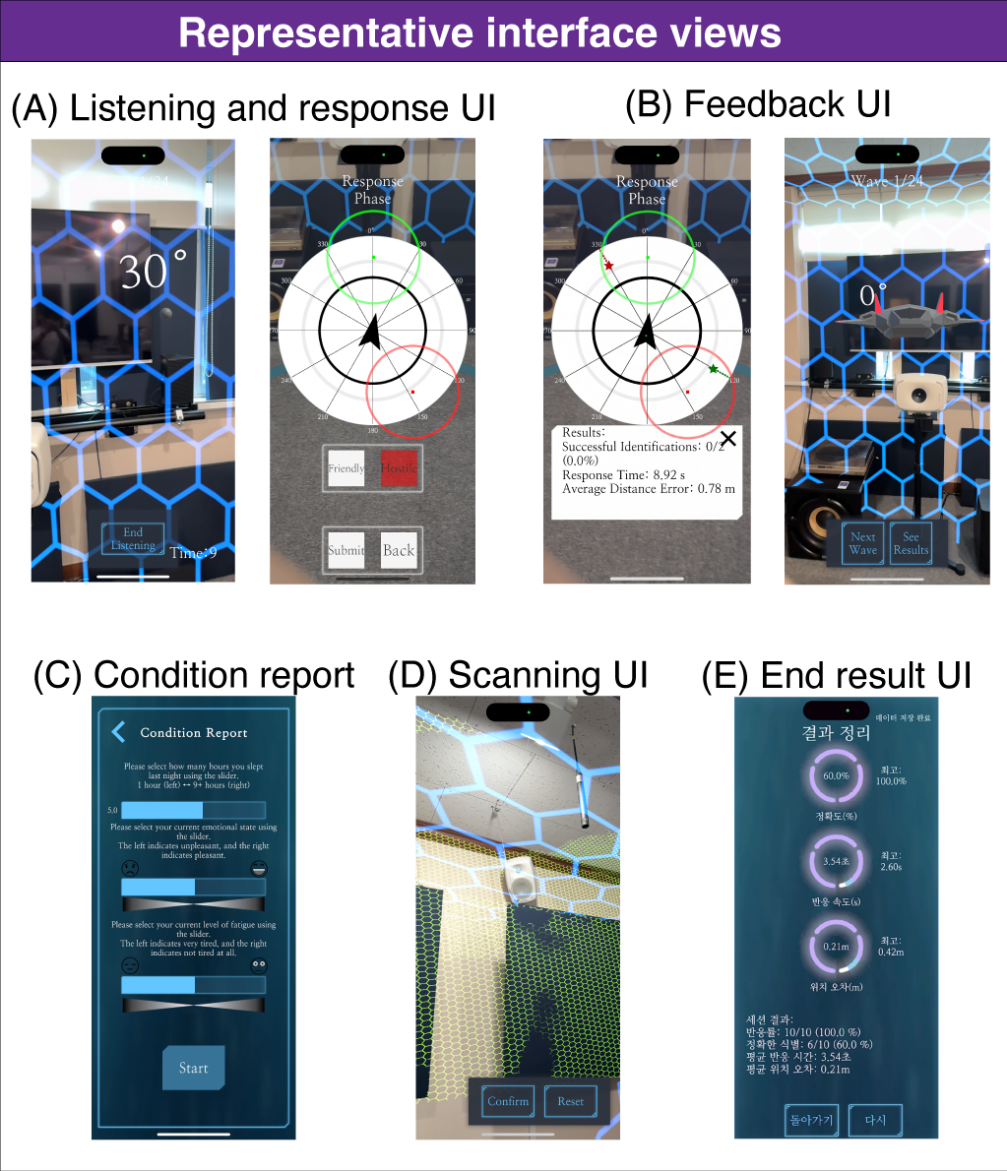
Representative interface screenshots illustrating key phases of the Augmented Reality Immersive Auditory training (ARIA) system. Screenshots were captured on the study-provided hardware (iPhone 14 Pro) and are illustrative of the app interface rather than being taken from participant sessions. UI: user interface.

Following setup, participants engaged in 36 training trials. During each trial, participants localized and discriminated two concurrent sound sources: 1 “hostile” and 1 “friendly,” consisting of distinct filtered noise bursts presented against continuous nonspatialized multitalker speech babble. For 20 seconds, players actively explored the soundscape by physically moving and rotating their heads to optimize spatial cues. Following this listening period, a 2D representation of the play area appeared on-screen, where players identified both the location and type of each sound source (Figure 2A: Listening & Response UI). After each response, participants received immediate feedback via a user interface display and AR spatial visualization showing correct source locations for errors. Including environment setup and condition reporting (~3 min) and active gameplay (~20 min depending on individual response speed), the total session duration was approximately 20 to 25 minutes.

Participants completed a 4-week protocol consisting of 4 training sessions and 1 evaluation session per week. Each session contained 36 trials. Training sessions provided immediate visual and auditory feedback to reinforce correct performance and narrative engagement. In contrast, weekly evaluation sessions withheld feedback to assess skill consolidation. To ensure the training was appropriately challenging, ARIA used an adaptive difficulty progression with 6 levels that varied systematically in acoustic complexity: changing the stimuli from stationary (fixed in 1 location) to moving (following a set path), decreasing the signal-to-noise ratio (SNR), and narrowing the correct answer threshold. All participants began at level 1, and subsequent levels were unlocked only after achieving 75% or more accuracy on the current level, allowing participants to advance at their own pace. The details of the level progression are outlined in Table 1.

**Table 1.** Augmented Reality Immersive Auditory training (ARIA) level difficulty parameters used in the 4-week at-home training protocol with middle-aged adults (N=11).

Level	SNR^a^ (dB)	Auditory stimuli	Correct threshold (m)
1	0	Stationary	1.0
2	0	Moving	1.0
3	−3	Stationary	1.0
4	−3	Moving	1.0
5	−6	Stationary	0.75
6	−6	Moving	0.75

a SNR: signal-to-noise ratio.

#### Deployment Context

The prototype developed for this study is not yet publicly available and was manually installed by the research team on all study devices. All participants were provided with identical devices (iPhone 14 Pro and Apple AirPods Pro 2) with preconfigured account credentials, eliminating the need for participant-managed login or setup. In-game performance data were uploaded automatically after each session to a laboratory-managed cloud database using anonymized participant identifiers, with access restricted to the research team. No personally identifiable information was stored alongside performance data. The system remained stable throughout the study period, with no instances of failed data uploads, mislogged sessions, or participant-reported technical failures. No modifications were made to the app or training protocol during the study period.

### Study Procedure

The study was conducted over 8 weeks and consisted of a baseline assessment, a 4-week training period, and a follow-up retention assessment. At baseline (week 0), participants attended an in-person session where they received study devices (iPhone 14 Pro and AirPods Pro 2) and completed a 30-minute tutorial with research staff guidance. Baseline speech-in-noise perception was assessed using the Korean Matrix Sentence Test (KMST) at 3 fixed SNR conditions (0 dB, −6 dB, and −9 dB) delivered diotically through Sennheiser HD 650 headphones at each participant’s most comfortable listening level in a sound-treated room [37]. Hearing thresholds were screened using the mobile Mimi Hearing Test app, which has previously been found to produce results comparable to standard audiograms [38]. Additionally, to account for potential baseline differences in auditory processing skills, participants’ musical experience was quantified using the Goldsmiths Musical Sophistication Index [39,40].

During the 4-week training period (weeks 1‐4), participants completed training and evaluation sessions at home on self-selected schedules. The research team sent scheduled reminders about remaining sessions and current progress. Participants returned for posttraining assessment at week 4, where the KMST was readministered using identical procedures, and participants completed the System Usability Scale (SUS) and Player Experience of Need Satisfaction (PENS) questionnaire, followed by a 20- to 30-minute semistructured exit interview [41,42]. A final follow-up at week 8 consisted of a KMST reassessment to evaluate retention of training effects.

### Outcome Measures

Speech-in-noise perception was measured using the KMST, with the percentage of correctly identified words at 3 SNRs (0 dB, −6 dB, and −9 dB) as outcomes. The ARIA system logged trial-by-trial performance, including localization accuracy (identifying sound source position), overall accuracy (both source position and discrimination), response time, and distance error. These metrics were analyzed separately for training sessions and evaluation sessions. User experience was evaluated through the SUS (0‐100 scale) and PENS, which assessed game engagement through Competence, Autonomy, and Presence subscales (1‐7 scale), with the Relatedness subscale omitted as ARIA lacks social features and nonplayable characters. Semistructured exit interviews were conducted to explore participants’ experiences with the AR technology, their perceived benefits and challenges, and their suggestions for improvement (Multimedia Appendix 2). Interview data were analyzed using reflexive thematic analysis. The lead author (PK) performed initial line-by-line inductive coding, followed by a process of collaborative refinement with the research team (SYK, HJL, and IYC). This peer debriefing allowed for a multiperspective reading of the data to establish the credibility and dependability of the thematic structure. All themes remained grounded in specific participant excerpts to maintain confirmability, while transferability was supported through the detailed reporting of our recruitment context and demographics [43,44].

All analyses were conducted using R version 4.3.1 (R Foundation for Statistical Computing) with an α level of .05. KMST improvements were assessed using paired 2-tailed *t* tests with Bonferroni correction for multiple comparisons across 3 SNR conditions. Effect sizes were calculated using Cohen *d*. Spearman correlations examined relationships between in-game performance improvements and KMST gains. Linear mixed-effects models assessed session-by-session learning trajectories for in-game metrics during evaluation sessions.

## Results

### Participant Characteristics and Feasibility

A total of 11 participants (8 males and 3 females) with a mean age of 53.0 (SD 3.0) years completed the 8-week study protocol. Demographic details, including education level, living conditions, usage of smartphones and video games, are summarized in Table 2. All participants who enrolled completed all phases of the study, resulting in a retention rate of 100%. Initial performance on the KMST at baseline showed a mean score of 92.5% (SD 2.3) at 0 dB SNR, 72.9% (SD 8.7) at −6 dB SNR, and 46.6% (SD 15.2) at –9 dB SNR. These 3 conditions represent graded levels of difficulty for the same speech-in-noise perception outcome, with lower SNRs imposing progressively greater perceptual and cognitive demands. Baseline scores confirmed that the more challenging conditions provided sufficient room for improvement while the near-ceiling performance at 0 dB SNR reflected participants’ functional hearing status.

**Table 2.** Participant demographics and characteristics (N=11).

Demographics and characteristics	Value, n (%)
Age (years)	
50-60^a^	11 (100)
Sex	
Male	8 (72.7)
Female	3 (27.3)
Education level	
High school graduate	1 (9.1)
Bachelor’s degree	5 (45.5)
Graduate degree	5 (45.5)
Household type	
Single person	1 (9.1)
With spouse	5 (45.5)
With spouse and children	5 (45.5)
Musical training	
None	10 (90.9)
Musically trained	1 (9.1)
Daily smartphone usage (hours)	
<1	3 (27.3)
1‐3	5 (45.5)
3‐5	3 (27.3)
Game experience^b^	
Nongamer	5 (45.5)
Casual gamer	4 (36.4)
Frequent gamer	2 (18.2)

a mean 53.0 (SD 3) years.

b Gaming experience was categorized based on self-reported frequency: "nongamer" indicates no play, "casual gamer" indicates monthly or biweekly play, and "frequent gamer" indicates weekly or daily play.

### Speech-in-Noise Perception (KMST) Improvements

**Figure 3.**
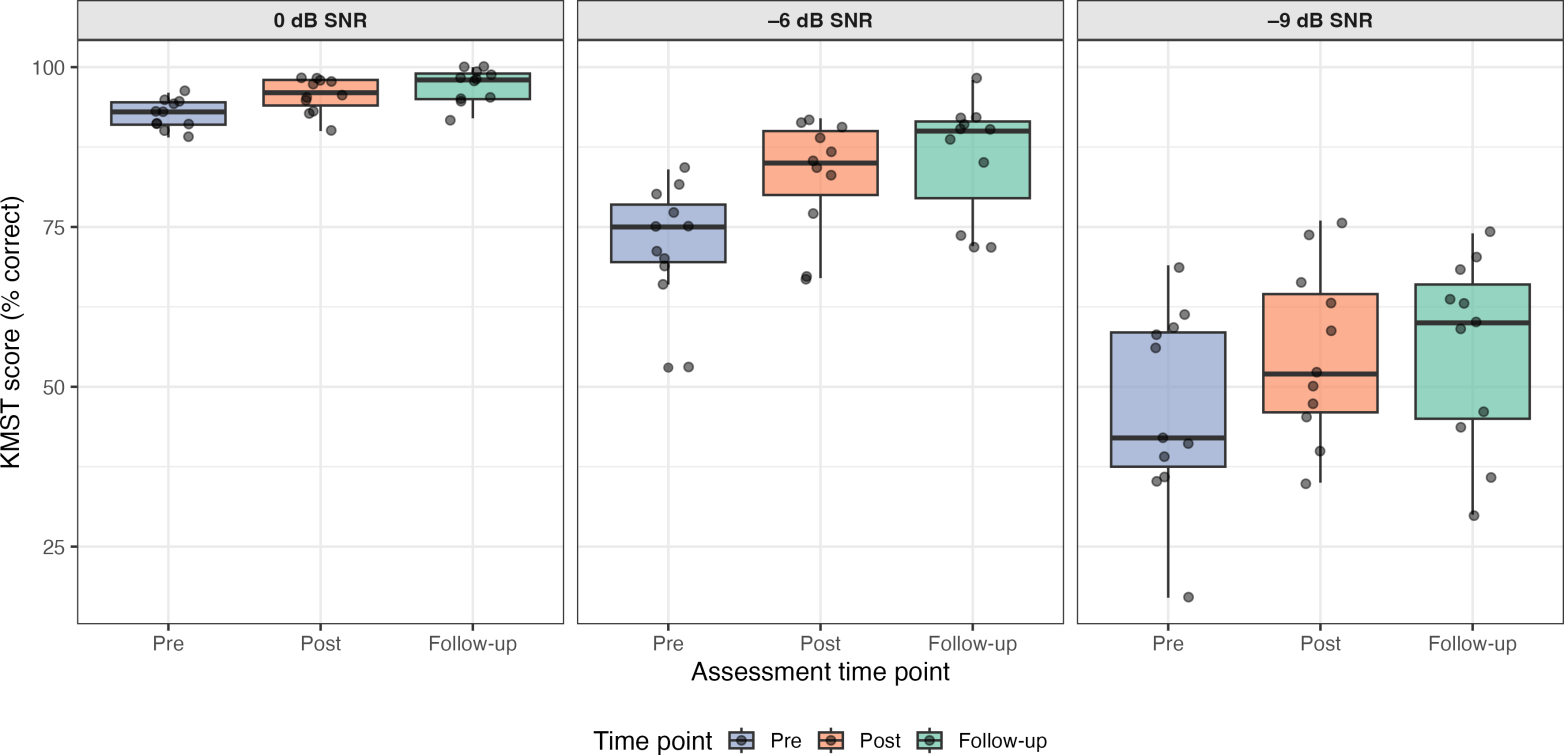
Speech-in-noise perception across study phases (N=11). Korean Matrix Sentence Test (KMST) scores at pretraining, posttraining, and 8-week follow-up, faceted by signal-to-noise ratio (SNR) conditions (at 0 dB, −6 dB, and −9 dB), representing graded levels of listening difficulty. Box plots display the median (IQR) and individual participant scores. Higher scores indicate better performance.

To evaluate the preliminary efficacy of the ARIA training program, exploratory analyses of speech-in-noise perception across all tested conditions were conducted. Paired 2-tailed *t* tests with Bonferroni correction yielded significant gains from pretraining to posttraining at 0 dB SNR (*t*_10_=3.43; *P*=.02; *d*=1.20),−6 dB SNR (*t*_10_=5.34; *P*<.001; *d*=1.14), and −9 dB SNR (*t*_10_=4.34; *P*=.004; *d*=0.58; Figure 3). Improvements were largest at the more challenging listening conditions, where baseline performance left greater room for change. All effect sizes met or exceeded the Cohen convention for medium effect (*d*>0.5); however, given the single-arm design and small sample size, these should be interpreted as preliminary efficacy signals. Individual participant analysis revealed consistent improvement patterns: 9 of 11 (81.8%) participants improved at 0 dB SNR, all participants improved at −6 dB SNR, and 10 of 11 (90.9%) participants improved at −9 dB SNR. All participants improved in at least 2 conditions. Follow-up assessment at 8 weeks showed no significant decline from posttraining scores at either −6 dB (*P*=.05) or −9 dB (*P*=.77), suggesting short-term retention. However, repeated exposure to the same test and the absence of a control group limit the interpretation.

### In-Game Learning Trajectories

**Figure 4.**
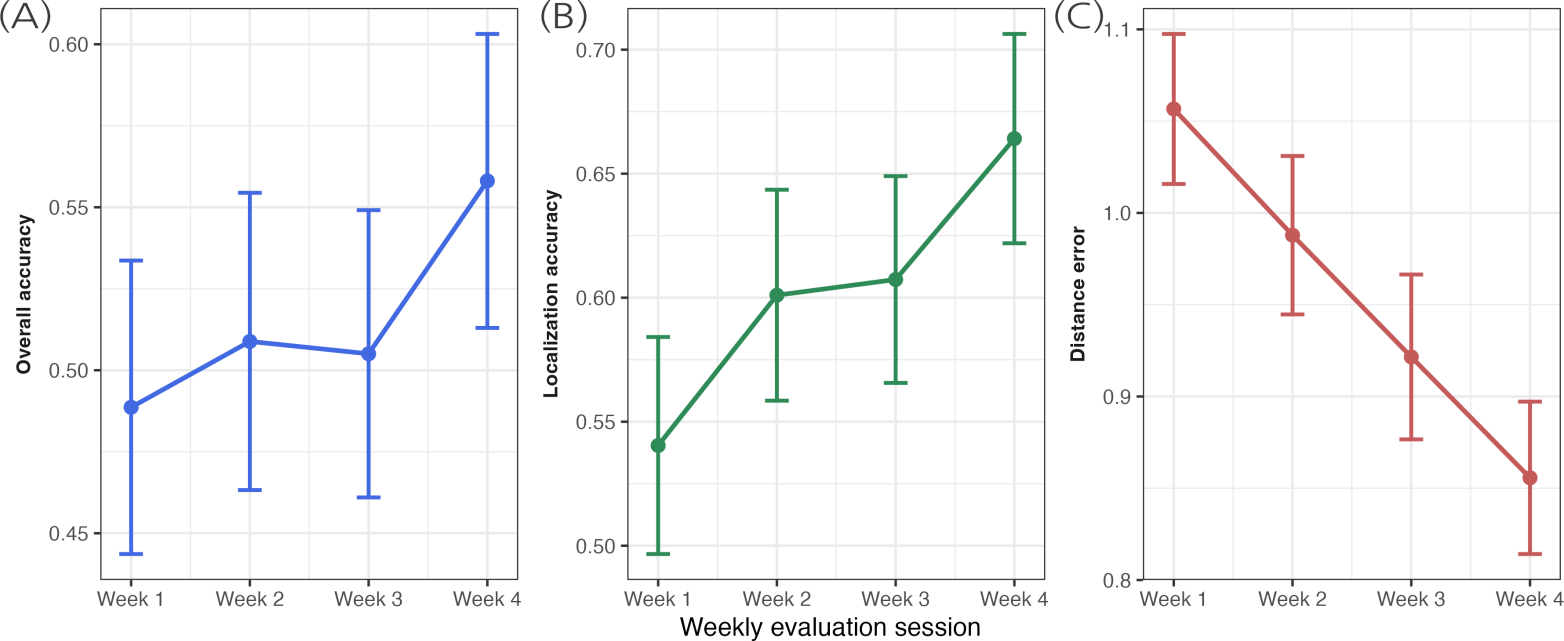
In-game skill improvement during evaluation sessions. Mean performance on key in-game metrics across the 4 weekly evaluation sessions, where no performance feedback was provided. (A) Overall accuracy (proportion of trials with correct sound identification and position). (B) Localization accuracy (proportion of correctly identified source positions). (C) Distance error between response and actual source position (meters; lower values indicate better performance). Error bars represent the SE of the mean.

Participants demonstrated significant learning within the ARIA game during weekly evaluation sessions. Level-matched analysis comparing the first and last evaluation sessions revealed robust improvements in foundational spatial hearing component skills: localization accuracy improved by 12.4% (*t*_10_=3.85; *P*=.003; *d*=1.16) and distance error reduced by 20.1% (*t*_10_=6.50; *P*<.001; *d*=1.96; Figure 4). However, overall accuracy—requiring simultaneous correct identification of both sound type and location—showed only a nonsignificant trend (6.9% gain; *t*_10_=1.58; *P*=.145; *d*=0.48). This dissociation suggests that while component spatial skills improved reliably, integrating these skills under the attentional demands of the full task remained more challenging. Linear mixed-effects models across all 4 evaluation sessions confirmed these patterns, with significant session-by-session improvements for localization (β=.038; *P*=.02) and distance (β=−.067; *P*<.001) but not overall accuracy (β=.020; *P*=.21). Presession self-report measures of sleep, fatigue, and valence did not show significant associations with in-game performance or learning trajectories, with individual response patterns suggesting limited variability across sessions.

### Transfer Effects: Skill-Specific Correlations

To explore potential mechanisms of improvement, correlations between in-game skill development and KMST gains were analyzed. Total training time showed a moderate positive correlation with KMST improvement at −6 dB SNR (ρ=0.547; *P*=.082), which approached but did not reach statistical significance.

Component skill analysis revealed stronger relationships for localization accuracy. Localization improvements during training correlated significantly with KMST gains at −6 dB SNR (ρ=0.639; *P*=.03) and −9 dB SNR (ρ=0.612; *P*=.045). Distance error reduction showed a weaker relationship with KMST gains at −6 dB (ρ=0.547; *P*=.08). These skill-specific correlations suggest a potential association between trained spatial hearing abilities and speech-in-noise performance, though the direction and underlying mechanisms of this relationship require confirmation in controlled trials.

### Usability and User Experience: System Usability and Engagement

The acceptability and usability of the AR-based intervention for the target population were assessed, as these factors are critical for determining real-world viability. The ARIA app demonstrated acceptable usability for the target population. The mean SUS score was 70.2 (SD 19.6), just above the industry definition of “average” usability [45]. More notable than the mean, however, was the wide range (47.5‐92.5), indicating substantial individual differences in usability perception that warranted further investigation through qualitative analysis. The PENS scale indicated that the core gameplay loop was engaging, with participants reporting high levels of Competence (mean 5.2, SD 0.89; range=3‐7) but more moderate levels of Autonomy (mean 4.5, SD 1.62; range=2‐5) and Presence (mean 4.8, SD 1.08; range=3‐6; Table 3). This pattern of high competence with moderate autonomy may reflect the structured training paradigm, where progression is prescribed rather than self-directed, and suggests that introducing greater player agency in session structure or difficulty selection could improve engagement.

**Table 3.** Usability and player experience scores (N=11).

Measure	Scale range	Mean (SD)	Median (range)
Usability			
System usability scale (SUS)	0‐100	70.2 (19.6)	72.5 (42.5‐95)
Player experience			
PENS: competence	1‐7	5.2 (0.89)	5.3 (5‐7)
PENS: autonomy	1‐7	4.5 (1.62)	5.0 (2‐5)
PENS: presence	1‐7	4.8 (1.09)	4.9 (3‐6)

### Qualitative Findings

#### Overview

To understand the drivers of the observed usability variance and to identify specific barriers to long-term adoption, a thematic analysis of exit interviews was conducted. Five themes emerged that contextualize the quantitative usability findings and provide actionable insights for intervention refinement.

Thematic analysis of the 11 participant interviews revealed 5 major themes regarding their experience with the AR Auditory Training Game. Four of these themes were highly dominant: (1) usability of AR and game setup, (2) attention and cognitive load, (3) the training experience as “homework,” and (4) enjoyment and motivation through mastery. A fifth theme, desire for multiplayer and social features, was also represented, mentioned by 45% (5/11) of participants. Interview durations were, on average, 23.1 (SD 2.1) minutes long.

#### Theme 1: Usability of AR and Game Setup

A highly dominant theme was the challenge participants faced with the game’s initial AR setup. Eight (72%) participants reported difficulties, primarily with the room scanning and space definition process, which they found to be inconsistent, time-consuming, and a recurring source of friction:

*The game itself once I had it setup was very simple and I got the hang of it after a couple of trials. But setting up the area to play? The part where I had to scan the room was very confusing, I did not know when to stop or if I did it correctly or not. After a while I got used to it, but I am still unsure if I did it well.*

[P03]

*When setting the location for the play area before playing...I realized that the degree markers were slightly different each time. Like the day before what was 0 degrees differed even though I set it at the same location. It was not consistent which made it confusing.*

[P10]

Several participants suggested that the process could be streamlined by allowing the game to save a previously scanned space, thereby removing the need to rescan for every session:

*The scanning itself I did not have too much difficulty getting used to [...] But honestly, it was annoying to set the same space for each session. I played at home and in the same location for all my sessions and a feature to save the space would be really helpful and save time.*

[P08]

#### Theme 2: Attention and Cognitive Load

All 11 participants identified that the game demanded a significant level of sustained attention and concentration. This was seen as the core cognitive skill required for success. The task became particularly demanding in higher-difficulty stages, where distinguishing between multiple, similar sounds added to the cognitive load:

*Listening to 2 sounds at once was a bit difficult...when I'm listening to one sound, it’s clear, but with two, I have to listen to this side and that side, and it gets confusing. Sometimes I even forgot which one was the hostile or friendly one as I was moving.*

[P02]

*You really need to be focused. When you're just a little distracted by something else, you miss it. Especially as the difficulty level is high, there’s almost no difference [between the sounds]...You have to be completely focused to catch the subtle characteristics.*

[P06]

This cognitive demand was also tied to physical and mental fatigue, with many noting that performance dropped when they were tired or when their concentration waned:

*The training while it was simple really demanded all my attention and on the days I had a tough day at work or slept too little. My performance dropped noticeably.*

[P05]

#### Theme 3: The Training Experience as "Homework"

The sentiment that the training felt like an obligation or “homework” was expressed by 9 (81%) of participants. This feeling was driven by the structured nature of the research, the required frequency of play, and the lengthy sessions, with a few exceeding 25 minutes, including the setup and condition report:

*To be honest, the feeling of it being a chore was stronger [...] Especially as I had to find time in my own life routine[...] During the weekdays I was only able to find time to do it after my work so I mostly did the training sessions at night.*

[P02]

The required duration of each session was a specific point of feedback related to this theme.

*Thirty six trials felt a bit long. My concentration would drop after about 15 to 20 trials and my performance really went down until like trial 30 when I realized I was almost done. I think having some way to pause the game so I could take a short break before continuing again would have helped a lot.*

[P09]

#### Theme 4: Enjoyment and Motivation Through Mastery

Despite the challenges, 7 (63%) participants found motivation and enjoyment in the process of improving and mastering the game. The positive feedback loop of successfully identifying a sound and seeing their accuracy scores increase was a primary driver of engagement. This sense of accomplishment was most potent when overcoming difficult levels:

*To pinpoint the exact moment where I found the game fun is difficult as the game itself was pretty simple and repetitive, but what I remember most is gradually getting more and more correct as I played… I could feel that my own skills were improving and which was the most satisfying feeling while playing the game.*

[P01]

*The most memorable moment was when I went to the higher levels. Because I was struggling at the earlier levels, it was intimidating to start the next level but even though I went to level 4 and I was still able to find them I knew I got a lot better which was rewarding.*

[P05]

The evaluation sessions, while sometimes frustrating, also served as a catalyst for motivation:

*While the first evaluation session was very difficult especially when I had to go through the levels that I did not try yet. The evaluation actually motivated me more. It made me want to get better quickly to challenge the next level and do better at the next evaluation session.*

[P07]

#### Theme 5: Desire for Multiplayer and Social Features

Several participants (5/11, 45%) suggested that adding multiplayer or social components would make the game more engaging and fun. The primary suggestion was to introduce a competitive element, such as leaderboards or head-to-head challenges, to foster motivation:

*Because I did the training at home, my family asked what I was doing and were interested in the game [...] If I could have played with them in some sort of competition maybe comparing scores or how fast we could find the sounds it would make it easier.*

[P02]

*If there was a system where you could see other people’s scores online or what level people are at, that would provide motivation...a little bit of a competitive spirit would kick in.*

[P03]

Cooperative gameplay was also mentioned, with 1 participant envisioning working together with family to complete the tasks:

*My kid loves playing games...I thought it would have been nice to play with him… just being able to spend time with him while doing something together, something simple like this game would be a good experience...It would be fun to find [the sounds] together.*

[P08]

## Discussion

### Principal Results

#### Overview

This formative pilot study evaluated the feasibility, acceptability, and preliminary efficacy of ARIA, a novel AR-based audio-motor training system for auditory training in middle-aged adults. Three primary aims were assessed. First, the study achieved 100% protocol completion over 8 weeks, demonstrating technical feasibility for at-home delivery; however, this retention rate was likely supported by performance-contingent financial compensation and may not generalize to nonincentivized contexts. Second, usability was acceptable (mean SUS=70.2, SD 19.6) but revealed substantial individual variability and critical barriers, particularly in AR environment setup. Third, exploratory efficacy analyses revealed preliminary signals of speech-in-noise improvement across all tested SNR conditions, with component skill improvements in localization correlating with KMST gains. Given the single-arm design and small sample, these findings warrant confirmation in controlled trials rather than interpretation as definitive intervention effects.

#### Critical Usability Barriers and Design Solutions

Thematic analysis revealed a stark dissociation between technical feasibility and user experience. Despite achieving 100% retention, 72% (n=8) of participants identified the AR environmental setup as confusing and time-consuming. This friction reflects a combination of current limitations in handheld AR environmental mapping technology and interface-level design issues within ARIA. Environmental mapping challenges, such as inconsistent scan results from day to day and misalignment of visual cues on the physical scene, are partially inherent to current consumer-grade mobile hardware. These represent a critical constraint that future development must address through interface-level innovations. Interface-level solutions, such as saved environments, real-time scanning feedback, and adjustable visual anchors, are immediately actionable and can help mitigate these hardware-level inconsistencies. This barrier aligns with broader literature documenting technology acceptance challenges among middle-aged and older adults using AR or VR systems [46]. The wide variance in the SUS scores (range 47.5‐92.5, SD 19.6) may partially reflect these setup difficulties, with setup friction predicting lower usability ratings. Participants’ suggestions converged on clear solutions: allowing the app to save previously scanned spaces to eliminate redundant scanning, providing clearer real-time feedback during scanning, adding adjustable visual indicators, and implementing a comparison of acoustics changed by the scanning process. Given that even this relatively young and technologically comfortable sample experienced substantial setup difficulties, these barriers may be more pronounced for older adult populations (aged ≥65 y) with less technology familiarity. However, because these barriers are identifiable, they represent addressable implementation constraints rather than fundamental limitations of the AR-based approach. Resolving these issues prior to deployment with older or clinical populations will be essential for scaling beyond controlled research settings.

#### Engagement Dynamics and “Homework” Perception

Despite setup frustrations, the core training paradigm demonstrated genuine engagement potential. PENS Competence scores averaged 5.2/7 with SD of 0.89, and 63% (n=7) of participants reported intrinsic motivation through mastery experiences, which aligns with research showing that middle-aged and older adults are primarily motivated by meaningful learning and tangible skill development in serious games [47].

However, 81% characterized the experience as “homework.” Qualitative analysis suggests this perception was primarily driven by the mandatory training regimen and fixed scheduling required by the research protocol rather than the game design itself. Participants reported difficulty integrating the required frequency into their daily routines, often resorting to completing sessions at night after work when cognitive resources were already depleted. While some users noted that the session length and the 36 consecutive trials felt demanding, this burden was significantly compounded by the external obligation to maintain the protocol’s required frequency. Notably, participants who described the training as homework simultaneously reported genuine enjoyment through mastery,“motivat[ing] me more...made me want to get better quickly” [P07]. This suggests the core gameplay successfully fostered competence motivation even within a compliance-driven context, while real-world deployment with self-paced scheduling may reduce this perceived burden.

#### Evidence for Skill Acquisition and Transfer

Participants described the training as cognitively demanding, emphasizing that sustained attention was critical for successful performance. This is consistent with our design intent: the dual-task requirement engaged participants in effortful selective attention, characteristic of real-world listening environments.

During training, participants demonstrated learning in foundational spatial hearing component skills such as localization, though integrated performance requiring simultaneous accuracy on both subtasks of discrimination and localization showed more modest gains. Qualitative feedback identified a specific constraint: at higher difficulty levels, sound discrimination became unreliably difficult, with users reporting they could “accurately localize sounds but having to guess” on source identity. This suggests that acoustic discriminability at high noise levels, rather than dual-task learning capacity, may have limited integrated performance. Future iterations should refine sound selection criteria or provide additional acoustic differentiation at higher difficulty levels.

Localization improvements during training correlated significantly with KMST gains at −6 dB and −9 dB, suggesting a potential skill-specific association that warrants investigation in controlled trials. These exploratory correlations suggest a potential association between trained spatial hearing abilities and speech-in-noise performance, consistent with audio-motor training frameworks proposing that spatial hearing training may enhance speech-in-noise perception through strengthened selective attention mechanisms [10-14,28]. However, without targeted cognitive assessments, it is not possible to establish whether improvements reflect enhanced spatial attention, domain-general cognitive gains, or other mechanisms. Future studies should incorporate comprehensive cognitive assessments including spatial working memory, auditory selective attention, and executive function to clarify transfer pathways.

#### An Unexpected Benefit: Hearing Health Awareness

Beyond the intended training effects, 1 participant reported that gameplay prompted them to seek a clinical hearing assessment after realizing they were missing sounds that family members easily detected—a phenomenon they would have previously dismissed. While based on a single case, this health-seeking behavior illustrates a potentially valuable secondary function of performance-based interventions: heightening awareness of one’s own capabilities through direct, experiential feedback rather than abstract self-evaluation.

This observation aligns with health belief model research identifying experiential “cues to action” as powerful predictors of health-seeking behaviors [48]. Interestingly, this awareness did not correlate with subjective ratings of real-world improvement, as participants felt that “daily life lacked the challenging noisy conditions of the game.” Rather than indicating transfer failure, this reveals an opportunity: ARIA successfully raised hearing capability awareness, but users lacked the context to recognize real-world applications of trained skills. Future versions could incorporate educational content explicitly connecting in-game performance to everyday listening scenarios, potentially serving dual purposes as both a training tool and a hearing health screening platform.

### Limitations

One limitation of this study is the absence of a control group and the small sample size (N=11), which together preclude definitive causal attribution. Observed KMST improvements may reflect practice effects, maturation, or placebo effects rather than training-specific benefits. While the short-term retention of gains at 8 weeks and the skill-specific correlations suggest a preliminary efficacy signal, only a randomized controlled trial with an active control can establish causality.

The assessment battery’s scope presents another key constraint. Our reliance solely on the KMST prevents conclusions about the cognitive breadth of training effects. Without targeted cognitive assessments, it is not possible to distinguish whether improvements reflect enhanced spatial selective attention—our proposed mechanism—or more general cognitive gains. Furthermore, the training occurred in varied home environments, where factors such as room geometry, lighting, and surface reflectivity were not modeled analytically, potentially influencing both user experience and performance outcomes.

Additionally, the financial compensation provided (up to ₩300,000, approximately US $216) may have influenced the 100% adherence rate and the reported “homework” sentiment. These results may not generalize to nonincentivized, real-world deployment contexts. Finally, sample homogeneity restricts generalizability. Participants were highly educated, technologically proficient, middle-aged adults (mean age 53.0, SD 3.0 y) with functional hearing. Findings may not generalize to older adult populations (aged ≥65 y) who may present with more advanced presbycusis, greater cognitive decline, and different technology adoption patterns. The current sample served as a feasibility testbed, but testing with older adult populations remains an essential next step. Additionally, participants had functional hearing not requiring amplification at baseline, whereas individuals with diagnosed hearing loss may show different training responses, engagement patterns, and usability challenges.

### Conclusions

This study demonstrated the feasibility of delivering a gamified, AR-based audio-motor training paradigm to middle-aged adults in their home environments. Beyond feasibility, several findings carry broader implications. The dissociation between successful core engagement and peripheral usability friction suggests that, for AR-delivered health interventions more generally, streamlining environmental setup is as critical as refining the intervention content itself. The exploratory correlations between spatial skill development and speech-in-noise gains provide a preliminary rationale for investigating audio-motor training as a mechanism for auditory intervention and potential rehabilitation. This hypothesis warrants further testing in controlled trials involving diverse clinical populations. Finally, the observed potential for performance-based training to promote hearing health awareness suggests a dual function for gamified auditory interventions—as both training tools and experiential screening platforms. These findings provide a foundation for refining the ARIA platform and designing a larger randomized controlled trial.

## Supplementary material

10.2196/91260 Multimedia Appendix 1 Gameplay demonstration video.

10.2196/91260 Multimedia Appendix 2 Interview guide questions.

## Abbreviations

AR: augmented reality
ARIA: Augmented Reality Immersive Auditory training
KMST: Korean Matrix Sentence Test
PENS: Player Experience of Need Satisfaction
SNR: signal-to-noise ratio
SUS: System Usability Scale
VR: virtual reality
