# Supplementary material for: An Augmented Reality Audio-Motor Training Game for Improving Speech-in-Noise Perception: Single-Arm Pilot Feasibility Study
Source: JMIR Form Res. 2026 May 14;10:e91260. doi: 10.2196/91260 (PMC13175522; doi:10.2196/91260)
Supplement: Multimedia Appendix 1 [file formative-v10-e91260-s001.docx]

Gameplay Demo Video Link:

https://youtu.be/vZDMnn_AnlA

Instruction Video Sent to Participants:

https://youtu.be/sinEMGxEC3o
