# Supplementary material for: An Augmented Reality Audio-Motor Training Game for Improving Speech-in-Noise Perception: Single-Arm Pilot Feasibility Study
Source: JMIR Form Res. 2026 May 14;10:e91260. doi: 10.2196/91260 (PMC13175522; doi:10.2196/91260)
Supplement: Multimedia Appendix 2 [file formative-v10-e91260-s002.docx]

Interview Guide

Translated Korean Questions to English

• When you first started using the AR game, were there any aspects did you found difficult or unfamiliar? If so, what were they?

• Were there any specific aspects of the gameplay controls that felt uncomfortable or difficult to understand? Please provide details.

• During the 4-week training period, were there any specific moments that were particularly memorable or enjoyable? (Please explain why you felt that way.)

• Do you feel that this game was helpful for your auditory perception in your daily life? If so, in which specific areas did you feel it helped (e.g., sound discrimination, concentration, etc.)?

• At what point during the 4-week training did you most feel like giving up? What motivated you to overcome that feeling and continue the training?

• Did you notice the difficulty level adjusting automatically as the training progressed? Do you think it would be more helpful if you could manually adjust the difficulty yourself?

• When conducting this training at home (or in a private space), how did your surrounding environment or the presence of others affect your concentration? (e.g., distractions from noise, encouragement from family members, etc.)

• What features would you like to see added to make the game more engaging over a longer period? (e.g., more diverse missions, new sound stimuli, or a reward system based on training performance.)

• If a 'new interaction method' were introduced to increase motivation, which of the following approaches do you think would be most effective for you?

- Training with others in real-time to feel a sense of belonging.
- Training individually, but sharing results or achievements later to validate progress.
- Performing cooperative missions where participants help or encourage each other.

• If you could share this training experience or participate with someone else, who do you think would have the most positive influence on your ability to continue training? (e.g., friends, family, or peers undergoing similar training.)

Original Korean Questions

• AR(증강현실) 게임을 처음 접하고 사용했을 때, 가장 어렵거나 낯설게 느껴졌던 부분은 무엇이었나요?

• 전반적인 게임 플레이 과정에서 조작이 불편하거나, 이해하기 어려웠던 점이 있었다면 구체적으로 무엇인가요?

• 4주간의 훈련 중 가장 기억에 남거나, 훈련이 즐겁다고 느꼈던 특별한 순간이 있었나요? (그렇게 느낀 이유도 말씀해 주세요.)

• 이 게임이 실제 청각 훈련에 도움이 되었다고 느끼시나요? 도움이 되었다면 구체적으로 어떤 부분(예: 소리 구분, 집중력 등)에서 그렇게 느끼셨나요?

• 4주라는 훈련 기간 중, 그만두고 싶다는 생각이 가장 많이 들었던 시점은 언제였나요? 그리고 그 시기를 넘기고 훈련을 지속하게 만든 원동력은 무엇이었나요?

• 훈련이 진행됨에 따라 난이도가 자동으로 조절되는 것을 체감하셨나요? 만약 본인이 직접 난이도를 설정할 수 있다면 훈련에 더 도움이 될 것 같나요?

• 이번 훈련을 집(혹은 개인적인 공간)에서 진행했을 때, 주변 환경이나 타인의 존재가 훈련 집중도에 어떤 영향을 미쳤나요? (예: 소음으로 인한 방해 혹은 가족의 응원 등)

• 향후 이 게임을 더 오랫동안 지루하지 않게 즐기기 위해 추가되었으면 하는 요소는 무엇인가요? (예: 더 다양한 미션, 새로운 소리 자극, 혹은 훈련 성과에 따른 보상 시스템 등)

• 만약 훈련의 동기부여를 높이기 위해 '새로운 상호작용 방식'을 도입한다면, 다음 중 어떤 방식이 본인에게 가장 효과적일 것 같나요?

- 다른 사람과 실시간으로 함께 훈련하며 소속감을 느끼는 것
- 각자 훈련하되, 나중에 결과나 성과를 공유하여 성취감을 확인하는 것
- 서로의 훈련을 돕거나 응원하며 협력하는 미션을 수행하는 것

• 만약 이 훈련 경험을 누군가와 공유하거나 함께 진행할 수 있다면, 누구와 함께하는 것이 훈련 지속에 가장 긍정적인 영향을 줄까요? (예: 친구, 가족, 혹은 비슷한 훈련을 하는 동료 등)
